# Preserved prenatal lung growth assessed by fetal MRI in the omicron-dominated phase of the SARS-CoV-2 pandemic

**DOI:** 10.1007/s00330-024-11031-9

**Published:** 2024-08-30

**Authors:** Gloria Biechele, Vanessa Koliogiannis, Philippe Rennollet, Tobias Prester, Enrico Schulz, Thomas Kolben, Magdalena Jegen, Christoph Hübener, Uwe Hasbargen, Andreas Flemmer, Olaf Dietrich, Tanja Burkard, Regina Schinner, Julien Dinkel, Maximilian Muenchhoff, Susan Hintz, Maria Delius, Sven Mahner, Jens Ricke, Anne Hilgendorff, Sophia Stoecklein

**Affiliations:** 1 Department of Radiology, University Hospital, LMU Munich, Marchioninistrasse 15, 81377 Munich, Germany; 2 Department of Obstetrics and Gynecology, Perinatal Center, University Hospital, LMU Munich, Marchioninistrasse 15, 81377 Munich, Germany; 3 Division of Neonatology, University Children’s Hospital, Dr. von Hauner Children’s Hospital and Perinatal Center, University Hospital, LMU Munich, Marchioninistrasse 15, 81377 Munich, Germany; 4 Max von Pettenkofer Institute and Gene Center, Virology, National Reference Center for Retroviruses, LMU Munich, Pettenkoferstraße 9a, 80336 Munich, Germany; 5 Department of Pediatrics – Neonatology, Stanford University, 750 Welch Road, Suite 315, Palo Alto, CA 94034 USA; 6 Institute for Lung Health and Immunity and Comprehensive Pneumology Center (CPC), Helmholtz Munich, Member of the German Lung Research Center (DZL), Max-Lebsche-Platz 31, 81377 Munich, Germany; 7 Center for Comprehensive Developmental Care (CDeCLMU), Dr. von Hauner Children’s Hospital, University Hospital, LMU Munich, Member of the German Lung Research Center (DZL), Lindwurmstraße 4, 80337 Munich, Germany; 8 Comprehensive Pneumology Center (CPC-M), Member of the German Center for Lung Research (DZL), Max-Lebsche-Platz 31, 81377 Munich, Germany

**Keywords:** Fetal MRI, Lung development, SARS-CoV-2, COVID, Omicron

## Abstract

**Objectives:**

With SARS-CoV-2 evolving, disease severity and presentation have changed due to changes in mechanisms of entry and effector site as well as due to effects of vaccination- and/or infection-acquired immunity. We re-assessed fetal lung pathology in pregnancies with uncomplicated SARS-CoV-2 infections during the late, omicron-dominated pandemic phase to inform disease understanding and pregnancy consultation.

**Methods:**

In this case-control study, fetal lung volumes were assessed by fetal MRI in 24 pregnancies affected by mild maternal SARS-CoV-2 infection during the omicron-dominated pandemic phase with prevailing immunity through vaccination and/or prior SARS-CoV-2 infection.

**Results:**

Fetal lung volumes (normalized to estimated fetal weight) in 24 pregnancies (GA 33.3 ± 3.8, 12 female fetuses) following mild, uncomplicated SARS-CoV-2 infection did not differ significantly from both, published reference values (96.3% ± 22.5% of 50th percentile reference values, *p* = 0.43), or fetal lung volumes of a site-specific, non-COVID control group (*n* = 15, 94.2% ± 18.5%, *p* = 0.76). Placental assessment revealed no group differences in thrombotic changes or placental heterogeneity (*p* > 0.05, respectively), and fetal lung volume did not correlate with placental heterogeneity when adjusting for gestational age at scan (*p* > 0.05).

**Conclusion:**

Assessment of fetal lung volume by MRI revealed unaffected lung growth in pregnancies affected by uncomplicated SARS-CoV-2 infection in the omicron-dominated pandemic phase in the presence of prevailing hybrid immunity. This finding contrasts sharply with the observed reduction in fetal lung volume following maternal alpha-variant infection in the pre-vaccination era and might reflect tropism- as well as immunity-related effects.

**Key Points:**

- *Question: Is fetal lung development affected by mild maternal SARS-CoV-2 infection during the omicron-dominated phase of the pandemic?*
- *Findings: Fetal lung volume in 24 affected pregnancies did not differ significantly from published reference values or fetal lung volumes in 15 site-specific, non-COVID-affected control pregnancies*.
- *Clinical relevance: Preserved fetal lung volume following mild maternal SARS-CoV-2 infection during the omicron-dominated phase contrasts with previous findings of reduced volume in unvaccinated pregnancies during the alpha-dominated pandemic phase. These observations might reflect tropism- as well as immunity-related effects*.

## Introduction

Fetal magnetic resonance imaging (MRI) has revealed a reduction in fetal lung volume in the offspring of women who experienced mild SARS-CoV-2 infection during the alpha-dominated, early phase of the pandemic [1]. With the virus evolving, it became clear that mechanisms of entry and effector site differed in virus variants of SARS-CoV-2, partially due to differential tropism-related effects [2–4]. Specifically, the omicron variant B1.1.529 [5] was found to result in lower disease severity despite increased transmissibility [6, 7], potentially due to its less severe involvement of the lower respiratory tract [6–8], but more efficient replication in nasal and bronchial tissues [6]. These characteristics were held accountable for the reduction in mortality and morbidity [9], and a decrease in adverse perinatal outcomes as compared to pre-omicron variants [10, 11], including a lower risk of preterm birth and neonatal death [12]. At the same time, an ambitious vaccination policy in Germany, Europe, and worldwide, explicitly included pregnant and breastfeeding women in the official COVID-19 vaccination recommendations [13, 14]. Vaccination policies and single or multiple infections occurring in large parts of the population due to viral spread resulted in hybrid immunity as a common phenomenon in the general population.

While pulmonary and overall outcomes in diseased individuals were at least partially improved, data on pregnancy outcomes remained sparse [15]. Here, the importance of addressing fetal lung growth in infections during the omicron-dominated phase of the pandemic became evident, supported by prenatal MRI revealing less severe placental changes in omicron as compared to pre-omicron variant infections [16]. Recent studies discussed the contribution of placental infection [17–19] to viral transmission into the amniotic fluid [20], which could facilitate viral transmission to the fetal airways and lung parenchyma in utero.

We hypothesized that these changes would also reflect on fetal lung volume. In order to address the effects of SARS-CoV-2 infection during pregnancy on fetal pulmonary development in the late, omicron-dominated, phase of the pandemic when hybrid immunity was prevailing, we investigated MR-based fetal lung volume in comparison to age-adjusted reference values and non-COVID controls. Revisiting our results obtained during the early stages of the pandemic phase [1] might highlight important tropism and immunity-related effects.

## Materials and methods

This prospective cohort study was approved by the local institutional review board (ethical approval #LMU-207–33). Written informed consent was obtained from all study participants.

A total of 24 pregnant women with mild SARS-CoV-2 infection during pregnancy were included in our study between February 2nd, 2022 and February 17th, 2023, when omicron was the prevailing SARS-CoV-2 variant in Germany [21]. SARS-CoV-2 was diagnosed at symptom onset by PCR in *n* = 19 patients and by a rapid antigen test in *n* = 5 patients. Infection occurred during the 1st trimester in *n* = 2, during the 2nd trimester in *n* = 16, and during the 3rd trimester in *n* = 6 pregnancies. Median time between test and MRI was 74.3 days (range 12–153), median gestational age (GA) at MRI was 33.3 weeks (range 24–38). *N* = 20 women had received three doses of vaccination against SARS-CoV-2 prior to MRI, *n* = 2 women had received two doses, and *n* = 2 women were not vaccinated against SARS-CoV-2. Last vaccination was performed at a median of 171.0 days (range 34–386 days) prior to SARS-CoV-2 diagnosis.

All women experienced only mild symptoms including anosmia/hyposmia (29%), ageusia (33%), fatigue (92%), headache (63%), cold (54%), dry cough (67%), sore throat (67%), and body aches (67%) and were therefore classified as “mild” according to the severity classification of the National Institute of Health’s COVID-19 treatment guidelines [22]. One patient reported gestational diabetes as a pregnancy complication diagnosed prior to MRI. No further complications were reported. No patient had received medication during pregnancy that could have caused damage to the placenta or the fetus. None of the women had received steroid treatment as respiratory distress prophylaxis prior to MR imaging. All women were Caucasian, except for one Asian woman. For patient characteristics see Table 1.

**Table 1:** Patient characteristics

	Mean ± Standard Deviation; Median [Range] or numbers (*n*)
Maternal age (years)	33.1 ± 4.6; 32.5 [25–42]
Trimester of infection (*n*)	1st (*n* = 2); 2nd (*n* = 16); 3rd (*n* = 6)
GA at SARS-CoV-2 infection (weeks)	22.4 ± 6.9; 21.0 [12–34]
Trimester of MR imaging (*n*)	1st (*n* = 0); 2nd (*n* = 3); 3rd (*n* = 21)
GA at MRI	33.3 ± 3.8; 33.5 [24–38]
SARS-CoV-2 test (*n*)	PCR (*n* = 19), rapid antigen test (*n* = 5)
time between SARS-CoV-2 test and MRI (days)	74.3 ± 43.8; 75.5 [12–153]
Vaccination (*n*)	3 doses (*n* = 20), 2 doses (*n* = 2), not vaccinated (*n* = 2)
time between last vaccination and SARS-CoV-2 test (days)	167.6 ± 89.1; 171.0 [34–386]
time between last vaccination and MRI (days)	241.8 ± 100.8; 255.0 [84–402]
Maternal symptoms (*n*)	anosmia/hyposmia (*n* = 7), ageusia (*n* = 8), fatigue (*n* = 22), headache (*n* = 15), cold (*n* = 13), dry cough (*n* = 16), sore throat (*n* = 16), and body aches (*n* = 16)
Maternal comorbidities (*n*)	CLD *n* = 2 (bronchitis/asthma); hypothyroidism *n* = 2; β-thalassemia *n* = 1; endometriosis *n* = 1; malignoma *n* = 1 (melanoma, cured)
Smoking (*n*)	before *n* = 6, during *n* = 0 pregnancy
Neonatal outcome (*n* = 16)	GA at birth (weeks): 39.7 ± 1.4; 40.0 [37–42] Apgar 10 min: 9.9 ± 0.5; 10.0 [8–10] Pulse oximetry: 99.1 ± 0.9; 99.0 [97–100] Body weight at birth (g): 3324.7 ± 360.2; 3267.5 [2750.0–3980.0]

Numbers are mean and standard deviation, median, range or numbers (*n*)

*GA* gestational age, *MR* magnetic resonance, *CLD* chronic lung disease, *g* grams

Fetal lung volumes were compared to reference values (see below). In addition, fetal lung volume and placental measurements were compared to a local non-COVID control group consisting of *n* = 15 pregnant women (median GA at MRI 29.7 weeks, range 24–35 weeks, six female fetuses), who underwent fetal MRI between 2017 and 2019 prior to the emergence of SARS-CoV-2. As > 30% of women tested positive for SARS-CoV-2 during pregnancy in the omicron-dominated pandemic phase [23], we included non-COVID control cases from a pre-pandemic period in order to avoid the inclusion of undiagnosed SARS-CoV-2 cases as controls (false negatives). This control group was previously described in detail [1] and allows the comparison to our previous dataset obtained during the alpha-dominated pandemic phase. MRI in the control group was performed for clinical indications pertaining to central nervous system development including suspected agenesis of the corpus callosum (*n* = 2 cases), asymmetry of the lateral ventricles (*n* = 9 cases), arachnoid cyst (*n* = 2 cases), plexus cyst (*n* = 1 case), and megacisterna magna (*n* = 1 case).

Study and control groups were scanned using an identical imaging protocol. MRI was performed at 1.5 Tesla (Magnetom Aera, Siemens Healthineers) with an 18-channel body coil using the scan protocol and sequence parameters described in Table 2. During fetal MRI acquisition, a radiologist was present at the scanner to ensure immediate image quality control and repetition of sequences in the event of motion artifacts or incompleteness.

**Table 2:** Imaging protocol and sequence parameters

	Orientation	TE (ms)	TR (ms)	ST (mm)	Matrix	Resolution (mm)	Phase resolution (% of total)
**Fetal body** TRUFI T1w FLASH DWI (b50 + b400)	tra, sag, cor cor cor	1.85 4.60 69.00	4.53 160.00 5400.00	5.0 4.5 5.0	0/256/256/0 0/256/205/0 0/126/158/0	1.2 × 1.2 1.4 × 1.4 2.4 × 2.4	100 80 80
**Fetal brain** T2w TSE T2 HASTE GRE tra Head TIRM DWI (b0 + b600) T1w FLASH	tra, sag, cor tra, sag, cor tra cor tra tra	140.00 83.00 30.00 5.60 69.00 4.60	11,380.00 800.00 1650.00 13,270.00 5400.00 160.00	4.0 3.0 5.0 5.0 5.0 4.5	0/256/192/0 256/0/0/256 128/0/0/128 384/0/0/307 0/158/126/0 0/256/205/0	1.3 × 1.3 1.1 × 1.1 2.5 × 2.5 1.0 × 1.0 2.4 × 2.4 1.4 × 1.4	75 100 100 80 80 80

*VIBE* volume interpolated breathhold examination, *HASTE* half-fourier acquisition single-shot turbo spin echo, *DWI* diffusion weighted imaging, *TSE* turbo spin echo, *GRE* gradient echo, *TIRM* turbo-inversion recovery-magnitude, *TRUFI* true fast imaging with steady-state free precession, *ms* milliseconds, *mm* millimeters

**Fig. 1:**
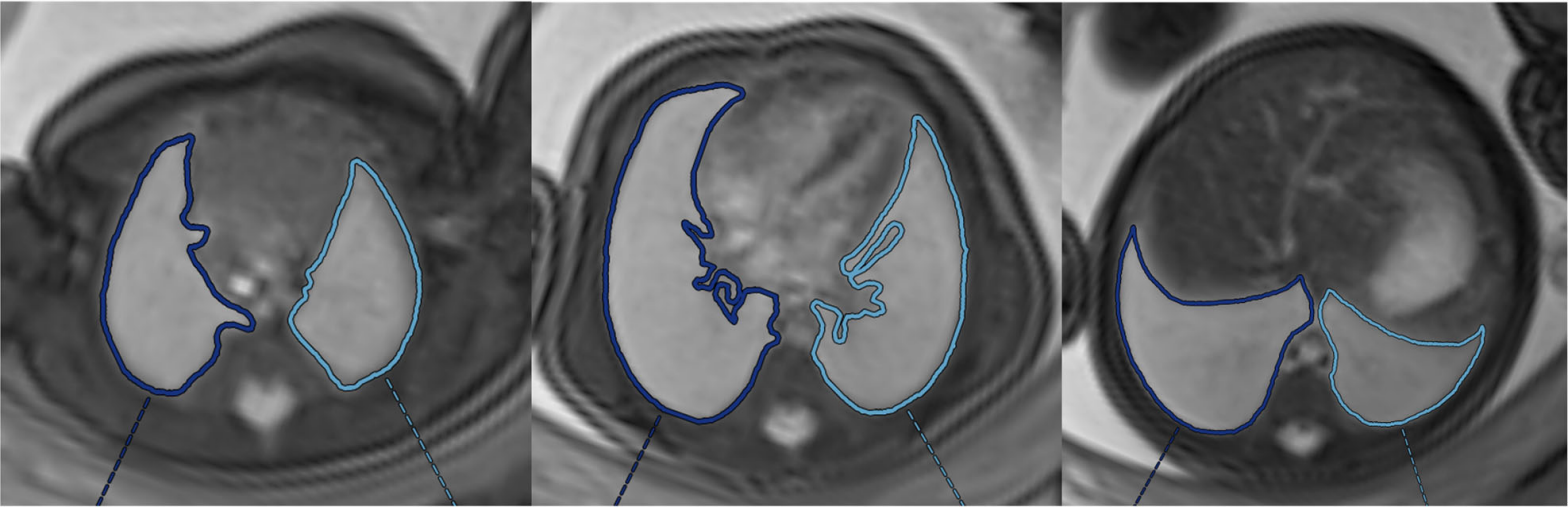
Segmentation of lung contours for lung volume estimation. Representative TRUFI (true fast imaging with steady-state free precession) images of the fetal lung in axial orientation at 36 weeks gestational age. Lung segmentation in three representative slices is shown in dark (right lung) and light (left lung) blue

Fetal lung volumes were determined independently by two board-certified radiologists with experience in fetal imaging. Fetal lungs were segmented on bright-fluid true fast imaging with steady-state free precession (TRUFI) images acquired in the fetal axial plane (Fig. 1). Lung images of the SARS-CoV-2-positive group and the non-COVID group were included in the reading sample. The reader was blinded to the diagnosis, as well as to all other clinical parameters such as time point and severity of infection. A second reader followed the identical protocol in order to assess inter-reader reliability using intraclass correlation.

Fetal body weight was estimated following a formula introduced by Shepard and colleagues [24] that was validated for MRI by Matthew and colleagues [25]: Fetal body weight (in kg) = 10 ^ (−1.7492 + 0.166 × BPD + 0.046 × AC) − (2.646 × (AC × BPD) / 1000); BPD = biparietal diameter, AC = abdominal circumference. Fetal lung volumes were normalized by estimated fetal weight and described as the percentage of the respective 50th percentile reference values [26].

$$\% \,{{{\rm{of}}}}\,{50}^{{{{\rm{th}}}}}\,{{{\rm{percentile}}}}\,{{{\rm{reference}}}}\,{{{\rm{value}}}}\,= \,\frac{{{{\rm{lung}}}}\,{{{\rm{volume}}}}/{{{\rm{estimated}}}}\,{{{\rm{body}}}}\,{{{\rm{weight}}}}}{{{{\rm{published}}}}\,{{{\rm{age}}}}-{{{\rm{matched}}}}\,{50}^{{{{\rm{th}}}}}\,{{{\rm{percentile}}}}\,{{{\rm{reference}}}}\,{{{\rm{value}}}}\,{{{\rm{of}}}}\,{{{\rm{lung}}}}\,{{{\rm{volume}}}}/{{{\rm{estimated}}}}\,{{{\rm{body}}}}\,{{{\rm{weight}}}}}$$

Reference values of age-adequate lung volume did not take fetal sex, maternal age, and race into consideration [26].

Placental heterogeneity and thrombosis were scored in a consensus reading by two board-certified radiologists with advanced experience in fetal and placental imaging. For the assessment of placental heterogeneity, we referred to the MRI-based placental grading scheme described by Blaicher and colleagues [27], which suggests four grades in accordance with the Grannum states. Grading ranged from 0, indicating no heterogeneity to 3, indicating severe heterogeneity. Grading of thrombotic changes ranged from 0, indicating no thrombotic changes to 3, indicating severe thrombotic changes.

Fetal lung volume, normalized to estimated fetal body weight, was described as the percentage of the respective 50th percentile reference values [26] and compared to a location mean of 1 using a signed-rank test. Group comparison between the SARS-CoV-2-positive group and the non-COVID group used Wilcoxon rank-sum test. Multivariable analyses of group effect (SARS-CoV-2-positive group versus non-COVID group), accounting for GA at scan and sex were performed using ANOVA. Within the SARS-CoV-2-positive group, multivariate analysis of the association between normalized fetal lung volume and GA age at scan, sex, trimester of infection, days between test and scan, and placental heterogeneity was also calculated using ANOVA. The normality of data was evaluated by visual inspection of histograms, QQ-plots, as well as tests for normality (Shapiro-Wilk, Kolmogorov-Smirnov, Cramer-von Mises, and Anderson-Darling). Placental changes were compared to the site-specific non-COVID group using Fisher’s exact tests.

The study was sufficiently powered to detect a reduction of lung volume to 85% (or less) of the expected reference value, which resembles the extent of lung volume reduction that we observed in our previous study during the early phase of the pandemic (80% power using a two-sided one-sample *t*-test at an alpha level of 0.05). Statistical analyses were performed using SAS Version 9.4 (SAS Institute Inc.).

## Results

### Preserved lung growth in fetuses following mild maternal SARS-CoV-2 infection in the omicron-dominated pandemic phase

**Fig. 2:**
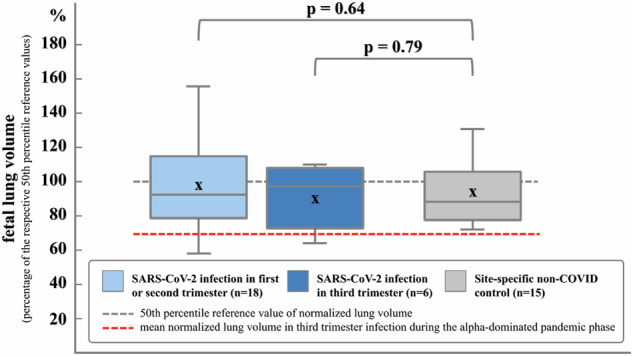
Normalized fetal lung volume stratified by trimester of infection. Boxplots of fetal lung volume normalized by estimated fetal body weight, expressed as a percentage of the respective 50th percentile reference values. Light blue indicates cases with SARS-CoV-2 infection during the omicron-dominated pandemic phase in the first (*n* = 2) and second (*n* = 16) trimester, dark blue indicates cases with infection in the third (*n* = 6) trimester. Gray indicates a site-specific non-COVID control group (*n* = 15). *p*-values are derived from group comparisons using Student’s *t*-tests. The gray dotted line indicates the 50th percentile reference value derived from Meyers et al [26], the red dotted line indicates the mean normalized lung volume as reported SARS-CoV-2 infection in the third trimester during the alpha-dominant pandemic phase [1]

The second reader confirmed fetal lung volume measurements showing excellent inter-reader reliability (ICC 0.97, Fig. S1). Lung volumes (normalized by body weight) in fetuses exposed to maternal SARS-CoV-2 infection during the omicron-dominant phase of the pandemic did not differ significantly from published reference values (96.3% ± 22.5% of 50th percentile reference values, *p* = 0.43). Likewise, when comparing the SARS-CoV-2-positive group to the site-specific non-COVID control group, no significant difference in normalized fetal lung volumes was observed (lung volume control group: 94.2% ± 18.5% of 50th percentile reference values, *p* = 0.76, Fig. 2). The group effect (SARS-CoV-2-positive group versus non-COVID control group) remained insignificant (*p* = 0.63), when adding sex (*p* = 0.11) and GA at scan (*p* = 0.58) to the statistical model.

Stratifying the results by trimester of infection (infection in the first or second trimester versus the third trimester) demonstrated no significant difference in comparison to published reference values (lung volume in the subgroup with infection in the first/second trimester: 97.8% ± 24.0%, *p* = 0.64, lung volume in the subgroup with infection in the third trimester: 91.8% ± 18.3%, *p* = 0.79), as well as in comparison to the site-specific non-COVID control group (*p* = 0.63 for the subgroup with infection in the first/second trimester, *p* = 0.79 for the subgroup with infection in the third trimester). In line with this, multivariate analyses detected no significant association of trimester of infection (*p* = 0.36), sex (*p* = 0.21), GA at scan (*p* = 0.72), or time between test and scan (*p* = 0.54) with normalized fetal lung volumes from SARS-CoV-2-positive pregnancies.

### No indication of placental pathology in pregnancies following mild SARS-CoV-2 infection in the omicron-dominated pandemic phase

On MRI, placental images in the SARS-CoV-2-positive group exhibited a comparable amount of thrombotic changes (*p* = 1.00) and placental heterogeneity (*p* = 0.061) when compared to placental tissue of the site-specific non-COVID control group (Table 3). There was no significant association between placental heterogeneity and normalized fetal lung volume within the SARS-CoV-2-positive group when taking GA at MRI into account (*p* = 0.16).

**Table 3:** Comparison of SARS-CoV-2-positive study group and non-COVID control group

	SARS-CoV-2-positive (*n* = 24)	non-COVID control group (*n* = 15)	*p*-value	statistical test
GA at MRI (weeks)	33.3 ± 3.8; 34 [24–38]	29.7 ± 3.4; 30 [24–35]	0.004*	Wilcoxon rank-sum test
fetal sex	*n* = 12 female (50%), *n* = 12 male (50%)	*n* = 7 female (47%), *n* = 8 male (53%)	1.0	Fisher’s exact test
fetal lung volume (mL)	83.1 ± 31.1; 88.6 [28.1–142.0]	53.2 ± 18.5; 54.0 [23.5–83.0]	0.003*	Wilcoxon rank-sum test
fetal lung volume (% of percentile)	96.3 ± 22.5; 92.8 [57.7–156.0]	94.2 ± 18.5; 88.7 [71.9–131.4]	0.762	Wilcoxon rank-sum test
fetal body weight (g)	2478.3 ± 835.1; 2498.5 [669.0–3640.0]	1703.2 ± 696.7; 1821.0 [582.3–2786.3]	0.005	Wilcoxon rank-sum test
placental heterogeneity (grades 0–3)	0: *n* = 8 (33%) 1: *n* = 11 (46%) 2: *n* = 5 (21%) 3: *n* = 0 (0%)	0: *n* = 1 (7%) 1: *n* = 6 (40%) 2: *n* = 8 (53%) 3: *n* = 0 (0%)	0.061	Fisher’s exact test
placental thrombotic changes	0: *n* = 19 (79%) 1: *n* = 5 (21%) 2: *n* = 0 (0%) 3: *n* = 0 (0%)	0: *n* = 12 (80%) 1: *n* = 3 (20%) 2: *n* = 0 (0%) 3: *n* = 0 (0%)	1.0	Fisher’s exact test

Numbers are mean and standard deviation, median, range or numbers and % of total

*GA* gestational age, *mL* milliliters, *g* grams

* Indicates *p* < 0.005, without adjustment for GA

### Uncomplicated postnatal adaptation following mild SARS-CoV-2 infection during pregnancy in the omicron-dominated pandemic phase

In *n* = 16 infants (mean GA at birth 39.7 ± 1.4 weeks, median 40.0 weeks, range 37–42 weeks) of the group exposed to mild maternal SARS-CoV-2 infection during pregnancy, uncomplicated postnatal adaptation without indication of acute postnatal respiratory distress was observed: 10 min Apgar score mean 9.9 ± 0.5, median 10.0, range 8–10, pulse oximetry mean 99.0% ± 0.9%, median 99.0%, range 97–100%. Median birth weight ranged from 2750 g to 3980 g (mean birth weight 3324.7 g ± 360.2 g, median 3267.5 g, range 2750.0–3980.0 g), with no infant ranging below the 5th percentile reference value. For detailed results of lung volume segmentation, fetal body weight estimation and placental reading please refer to Table 3 and Fig. S2.

## Discussion

Using fetal MRI, we demonstrated preserved fetal lung growth following mild SARS-CoV-2 infection during pregnancy in the late, omicron-dominated phase of the pandemic. This finding is in stark contrast to data obtained in pregnancies following mild SARS-CoV-2 infection during the early, alpha-dominated, pre-vaccination phase of the pandemic where fetal MRI revealed a significant reduction in fetal lung volume, especially when infection had occurred in the third trimester [1]. In a combined analysis of both our data sets, we found a significant group effect (alpha- versus omicron-dominated) on normalized fetal lung volume (*p* = 0.04).

Our findings reflect on several phenomena that impact on virus-host interaction. First, the evolved tropism of the omicron variant, which was shown to replicate predominantly in the epithelium of the upper airway and less efficiently in the lung parenchyma [6, 7], accounts for differences when compared to the alpha SARS-CoV-2 variant [4]. The reduced affinity to alveolar type II cells might limit the virus’ effects on the developing fetal lung, thereby ameliorating the impact on lung growth and volume expansion. Second, data obtained from the omicron-dominated pandemic phase demonstrated reduced affinity of the virus to the placenta as compared to other SARS-CoV-2 variants [28, 29], potentially resulting in a reduced rate of (transient) exposure of the fetal lung to the virus due to preserved placental integrity as well as a reduced rate of indirect, placenta-related effects of the infection. In pre-omicron variants, proof of the virus in the placenta was found in 21% of SARS-CoV-2 positive pregnancies, demonstrating infection and resulting in consecutive pathologic changes including fetal vascular malperfusion (35.3%), villitis (8.7%), intervillositis (5.3%), and chorioamnionitis (6%) [30], potentially affecting fetal development. Less placental pathology in omicron compared to pre-omicron infected pregnancies has been shown in a recent MRI study [16], and is further confirmed by the findings of this study, which did not reveal a significant difference in placental heterogeneity and thrombosis between the SARS-CoV-2-positive group and the non-COVID control group. Preserved placental integrity might limit direct and indirect effects on the fetal lung.

Third, and side-by-side with tropism-related effects, our findings likely reflect the effects of acquired immunity. In contrast to the early pandemic phase, where vaccination was not yet available or recommended for pregnant women in Germany [13], the late pandemic phase was characterized by prevailing hybrid immunity, with the majority of the population in Germany having undergone vaccination as well as single or multiple SARS-CoV-2 infections. Immunity through vaccination has been shown to ameliorate SARS-CoV-2 effects in pregnant women and their offspring, with reduced risk for severe maternal, neonatal, and perinatal morbidity and mortality [31], including reduced risk of stillbirth and prematurity for neonates [32]. While studies indicated a stronger immune response following vaccination as compared to natural infection [33], hybrid immunity resulted in a more effective and longer-lasting protection against severe infection-related morbidities [34].

While making a strong case on pregnancy outcomes in the late omicron and hybrid-immunity-dominated phase of the pandemic to be used for counseling pregnancies, our data necessarily include certain limitations, partially pertaining to limited sample size: First, our dataset is not suited to disentangle effects of viral variant and effects of acquired immunity. While infections during pregnancy were proven by PCR (*n* = 19) or antigen testing (*n* = 5), antibody testing to detect previous infections as well as assessment of viral load were not performed. As viral load was not shown to be associated with placental transmission [35] or main perinatal outcome parameters such as birth weight or prematurity [36] and as only 1% of neonates born to infected mothers tested positive for SARS-CoV-2 emphasizing postnatal rather than vertical infection [37] while not ruling out transient or local fetal infections, we did not perform tests for viral infection on the fetus or neonate in order to avoid additional blood draws or amniocentesis. Further, although our result of preserved fetal weight is in line with publications demonstrating unchanged fetal growth during SARS-CoV-2 infections [38, 39], it has to be taken into account that the MRI-based assessment of fetal body weight was validated only for the 2nd trimester [25], despite its original development based on ultrasound data of all three trimesters [24].

Despite the lack of early effects on fetal lung growth following mild maternal SARS-CoV-2 infection during the omicron-dominated pandemic phase, maternal infection might have long-term implications for the pulmonary health of the offspring [40]. This important aspect will be addressed in a longitudinal assessment following the patients of this study until five years of age.

Our finding of preserved fetal lung volume following mild maternal SARS-CoV-2 infection during the omicron-dominated pandemic phase with prevailing hybrid immunity provides insight into potential similarities of viral “behavior” as our findings mirror the omicron-characteristic respiratory findings in adults. Realistically capturing current disease constellations, our findings can inform counseling of women affected by mild SARS-CoV-2 infection during pregnancy. The study furthermore highlights fetal MRI as a useful tool to assess environmental as well as population health-related effects on fetal lung development. Future studies are needed to assess the long-term effects on lung health in this vulnerable patient population following SARS-CoV-2 infection pre-birth.

## Supplementary information

ELECTRONIC SUPPLEMENTARY MATERIAL

## Abbreviations

GA: Gestational age
MRI: Magnetic resonance imaging
TRUFI: true fast imaging with steady-state free precession
