# Supplementary material for: Preserved prenatal lung growth assessed by fetal MRI in the omicron-dominated phase of the SARS-CoV-2 pandemic
Source: Eur Radiol. 2024 Aug 30;35(3):1514–21. doi: 10.1007/s00330-024-11031-9 (PMC11835889; doi:10.1007/s00330-024-11031-9)
Supplement: Supplementary file 1 — ELECTRONIC SUPPLEMENTARY MATERIAL [file 330_2024_11031_MOESM1_ESM.pdf]

Preserved prenatal lung growth assessed by fetal MRI in the omicron-dominated phase of SARS-CoV-2 pandemic

ELECTRONIC SUPPLEMENT

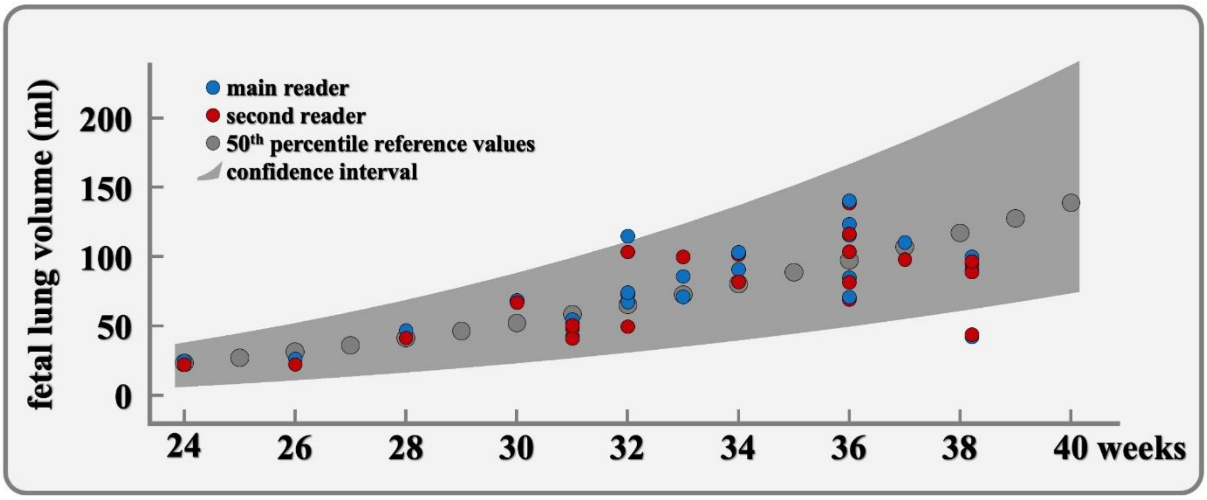

ARY MATERIAL

Supplemental Figure 1: Fetal lung volume over gestational age at scan

Gray dots indicate 50th percentile reference values of fetal lung volume (ml) over gestational age (weeks), gray area indicates 5% to 95% confidence interval of reference values. Blue dots indicate fetal lung volumes (ml) of SARS-CoV-2-positive group estimated by the main reader, red dots indicate fetal lung volume (ml) estimated by the second independent reader (n=24, respectively). One fetus at 38 weeks displayed low total lung volume in both independent readings and corresponding low estimated fetal body weight (see supplemental Figure 2).

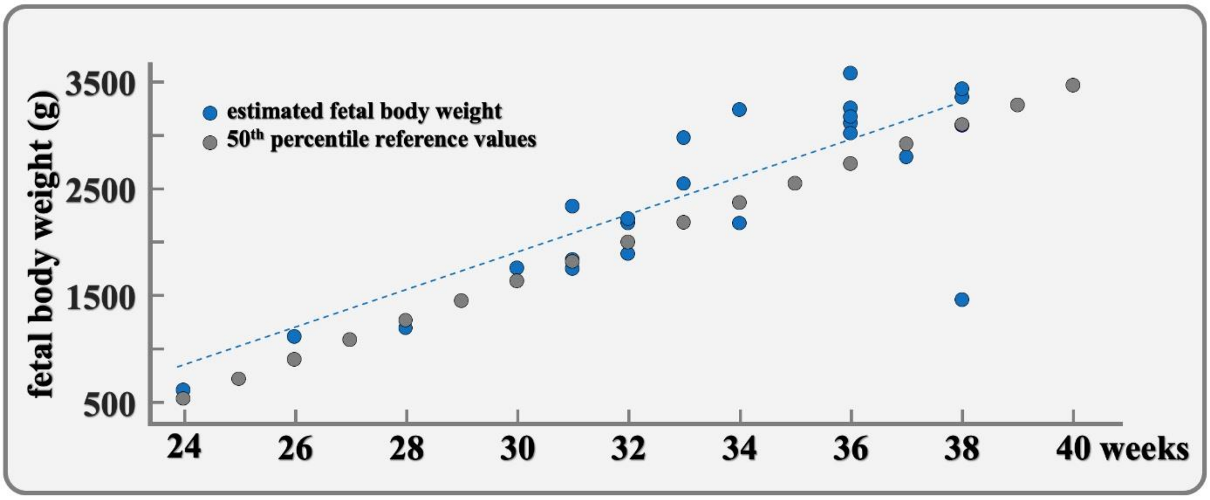

Supplemental Figure 2: **Fetal body weight over gestational age at scan**

Gray dots indicate 50th percentile reference values of estimated fetal body weight (g) over gestational age (weeks) and blue dots indicate estimated fetal body weights (g) in the SARS-CoV-2-positive group (n=24).
